# The Effect of Salience on Chinese Pun Comprehension: A Visual World Paradigm Study

**DOI:** 10.3389/fpsyg.2020.00116

**Published:** 2020-02-04

**Authors:** Wei Zheng, Yizhen Wang, Xiaolu Wang

**Affiliations:** ^1^School of International Studies, Zhejiang University, Hangzhou, China; ^2^School of Foreign Language Studies, Ningbo Institute of Technology, Zhejiang University, Ningbo, China; ^3^School of Humanities and Communication Arts, Western Sydney University, Penrith, NSW, Australia

**Keywords:** Chinese pun comprehension, salience effect, graded salience hypothesis, direct access model, visual world paradigm

## Abstract

The present study adopted the printed-word visual world paradigm to investigate the salience effect on Chinese pun comprehension. In such an experiment, participants listen to a spoken sentence while looking at a visual display of four printed words (including a semantic competitor, a phonological competitor, and two unrelated distractors). Previous studies based on alphabetic languages have found robust phonological effects (participants fixated more at phonological competitors than distractors during the unfolding of the spoken target words), while controversy remains regarding the existence of a similar semantic effect. A recent Chinese study reported reliable semantic effects in two experiments using this paradigm, suggesting that Chinese participants could actively map the semantic input from the auditory modality with the semantic information retrieved from printed words. In light of their study, we designed an experiment with two conditions: a replication condition to test the validity of using the printed-word world paradigm in Chinese semantic research, and a pun condition to assess the role played by salience during pun comprehension. Indeed, global analyses have revealed robust semantic effects in both experimental conditions, where participants were found more attracted to the semantic competitors than to the distractors with the emergence of target words. More importantly, the local analyses from the pun condition have shown that the participants were more attracted to the semantic competitors related to the salient meaning of the ambiguous word in a pun than to those related to the less salient meanings within 200 ms after target word offset. This finding suggests that the salient meaning of the ambiguous word in a pun is activated and assessed faster than its less salient counterpart. The initial advantage observed in the present study is consistent with the prediction of the graded salience hypothesis rather than the direct access model.

## Introduction

Although linguistic ambiguity seems harmful to the clarity of communication, it is not uncommon for people to intentionally exploit this uncertainty to achieve specific linguistic or rhetorical effects. Puns are one such case. Unlike temporary ambiguity, punning is a rhetorical device consisting of intentional exploitation of phonetic measures to evoke two meanings (Guidi, 2012). In the example used by Coulson and Severens (2007) “During branding, cowboys have sore calves,” quite intuitively, successful appreciation of the humorous effect of the pun requires one to access both the “cow” and “leg” meanings of the homograph *calf.* However, in contrast to the abundant literature on lexical ambiguity resolution, research on the lexical processing of puns is still far from enough, and it is still not entirely clear whether the two meanings of a pun are accessed sequentially or simultaneously. To shed more light on this issue, the current study aims to investigate the meaning activation process of a pun, and specifically to test whether this process is modulated by the saliency of the two meanings of the critical ambiguous word.

So far, two factors have been widely identified that influence lexical ambiguity processing: context and meaning frequency. Although researchers are generally in consensus that context plays a crucial role in distinguishing intended meanings, the dispute remains as to when context begins to exert its influence. The modular account claims that meaning activation of a lexical item is autonomous and immune to any contextual effect, while context only plays a selection role at the later integration stage (Fodor, 1983). The interactive account, on the other hand, argues that context can influence the lexical process at a very early stage and that the activation of context-incompatible meaning can be surpassed given sufficient contextual support (McClelland and Rumelhart, 1981). On the other side of the story, meaning frequency is assumed to compete with the contextual effect. Duffy et al. (1988, 2001) reported increased processing difficulties experienced by their participants while they were reading sentences biasing toward the less frequently used (subordinate) meaning of an ambiguous word. This so-called “subordinate bias effect” was then frequently taken as evidence for the meaning frequency advantage in the lexical resolution literature.

Up to now, various lexical ambiguity resolution models have been proposed, differing in how the two above-mentioned factors are taken into consideration, which sheds light on the investigation of pun comprehension (Wang and Zheng, 2019). Among them, two models are of particular interest for the current study, the direct access model (DAM) proposed by Glucksberg et al. (1986) and the graded salience hypothesis (GSH) by Giora (1997, 2003). DAM argues that context can interact with the initial lexical access to such an extent that the context-compatible meanings can be accessed directly without accessing other possible meanings if the context strongly biases toward that interpretation. However, GSH claims that contextual and lexical information is governed by two parallel systems: a predication-based central mechanism that can utilize both linguistic information and world knowledge, and a modular lexical mechanism that is only sensitive to information inside the mental lexicon. Upon encountering a word stimulus, the salient (more conventional, frequent, familiar, or prototypical) meaning is invariantly processed faster than the less salient ones. Even when the context strongly supports the less salient meaning, the salient meaning still cannot be surpassed.

When it comes to puns, where the two meanings of an ambiguous word (usually a homograph or homophone) are similarly supported by the pun context, the two models have different predictions. Specifically, DAM predicts simultaneous activation of both meanings while GSH predicts earlier access of the salient meaning than the less salient but context-compatible meaning. In the limited pun processing literature, however, both models seem to gain some support.

In the pioneering study on the meaning activation process during pun comprehension, Coulson and Severens recorded the event-related potentials (ERPs) while their participants were listening to pun sentences (e.g., *During branding, cowboys have sore calves*). A probe which was rated as highly related (e.g., *cow*), moderately related (e.g., *leg*) or unrelated to the pun (e.g., *calves*) was presented on either the left visual field (right hemisphere) or right visual field (left hemisphere) after the target word offset. It was found that both related probes elicited N400 priming effects (smaller N400 amplitude^1^) than the unrelated controls, but only the highly related probe yielded such effect in the right hemisphere in the short inter-stimulus interval condition (ISI = 0 ms). In contrast, N400 priming effects were found for the highly and moderately related probes in both hemispheres in the long inter-stimulus interval condition (ISI = 500 ms). These findings suggest that both meanings evoked by a pun are activated immediately in the left hemisphere, but only the highly related meaning is also activated in the right hemisphere. By 500 ms, however, both meanings are available in both hemispheres. Therefore, their results (simultaneous meaning activation in the left hemisphere and faster activation of salient meanings in the right hemisphere) provided partial support for both DAM and GSH.

Results from other studies, however, seem to be more consistent with GSH (Giora, 1997, 2003). In an eye-tracking study, Sheridan et al. (2009) asked their participants to read ambiguous words embedded in two types of sentence contexts. In the subordinate context, the less frequently used subordinate meanings are more plausible (e.g., *The man with the toothache had a* ***crown*** *made by the best dentist in town*). In the pun context, both meanings of the ambiguous words are equally possible (e.g., *The king with the toothache had a* ***crown*** *made by the best dentist in town*). Their results showed that fixation duration to the ambiguous word *crown* was longer in the subordinate context than that in the pun context, while the reverse pattern was observed in the following disambiguating region (e.g., *made by the best dentist in town).* They argued that the longer fixation duration in the subordinate context was an instantiation of the so-called subordinate effect (Duffy et al., 1988, 2001), where the less salient “dental device” meaning *crown* became more available and competed with the salient (dominant) “royal ornament” meaning in a similar time window, hence “reordering” the lexical access process. While in the pun context, no such “reordering” occurred since the balanced pun context could not reverse the frequency advantage of the salient meaning. In another ERP study, Dholakia et al. (2016) found that the pun context condition (e.g., *The prince with a bad tooth got a* ***crown***) elicited smaller N400 amplitudes (i.e., priming effect) on the homonyms than the neutral context condition (e.g., *The adult with a bad leg got a* ***crown***). However, the pun context did not elicit a larger N400 priming effect in comparison to a single dominant biasing context (e.g., *The prince with a bad leg got a* ***crown***). Like Sheridan et al. (2009), these authors attributed their findings to the tug of war between the superiority of salient (dominant) meanings and that of contextual support. Taken together, these two studies indicate that the two meanings of the ambiguous word in a pun are activated sequentially, lending support to GSH or the reordered access model in these authors’ words.

In respect of the conflicting findings, more empirical data are needed to test the two models and to further our understanding of the meaning activation process during pun comprehension. To this end, we have carried out the current study with two new perspectives. Firstly, we used Chinese puns as the experimental materials in the present study. Till now, the only few pun studies available in the psycholinguistic and cognitive literature have been almost exclusively on English, and relevant studies on Chinese puns can even hardly be found. Therefore, research on Chinese puns can contribute cross-linguistic data to the literature. Secondly, we used reaction time as the measurement to decide the saliency of different meanings associated with a homonym. According to GSH, salience is an end-product decided by factors like frequency, familiarity, conventionality, etc., among which familiarity usually has a larger weight. However, salience seems to be equated with frequency in some previous studies, ignoring other factors that also matter. Take *bank* for example, “financial institution” is usually deemed as its salient (dominant) meaning due to the higher frequency of use, while “riverside” as the less salient (subordinate) meaning. However, it is highly likely for a fisherman to come up first with its so-called subordinate “riverside” meaning when hearing the word *bank*. Although similar concern has been raised by Mchugh and Buchanan (2016), the lexical co-occurrence method used in their study is subjective to the same problem as using meaning frequency to decide salience. As a result, we followed the suggestion proposed by Giora (2003) and used reaction time to measure salience, which could better reflect the overall cognitive status of the participants’ population (see the detailed procedures in the “Materials and Methods” section).

In addition, we have adopted a printed-word version of the visual world paradigm (VWP) for the current study. In a traditional VWP experiment, participants are required to listen to a sentence while looking at a visual display of four pictures. By tracking eye movements, researchers can probe into the dynamic process of spoken language comprehension. It is reported that fixation proportions on different picture referents are modulated by the available phonological/orthographic, semantic, or even shape information while the spoken sentence is unfolding (Tanenhaus et al., 1995; Allopenna et al., 1998; Huettig and McQueen, 2007; see also Huettig et al., 2011 for a review). For example, Allopenna et al. (1998) presented the participants with a screen of four object pictures while they were listening to a sentence with the pattern “click on the *Target*.” They found a temporary increase in the fixation proportion to the onset-matched phonological competitors (e.g., *beetle*) when the target word (e.g., *beaker*) was heard, followed by a smaller increase in the offset-matched phonological competitors (e.g., *speaker*). The results were taken as evidence for the cohort meaning activation pattern predicted by continuous mapping models like TRACE (McClelland and Elman, 1986). In the two of a series of four experiments, Huettig and McQueen (2007) found similar results with a modified version of the traditional VWP, where the visually presented objects were replaced by the corresponding words. Based on their findings, they claimed that the printed-word version of VWP could also be used for studies involving phonological representations. However, they remained doubtful about its validity for studying questions involving semantic representations.

Since most of the previous VWP studies have been conducted with alphabetic language materials, an interesting question arises: whether semantic information could be utilized to such an extent that it can shift visual attention in such a paradigm when logographic languages (like Chinese) are used. To answer this question, Shen et al. (2016) asked their participants to listen to spoken Chinese sentences (e.g., *In Liberia, people often call* ***doctors*** *angels.*) while they were looking at a visual display of four 2-character words. These words include a semantic competitor (e.g., *nurse*-护士 hu4 shi4) which is semantically related to the spoken target word (e.g., *doctor*-医生/yi1 sheng1/), a phonological competitor whose first character shares the same sound and tone in Chinese pinyin (*hanger*-衣架/yi1 jia4/) with that of the target word, and two unrelated distractors (e.g., *school*-学校/xue2 xiao4/and *insect*-昆虫/kun1 chong2) which are neither semantically nor phonologically related to the target word. According to their results, the participants fixated more at semantic competitors than distractors both when the visual stimuli preceded target words for 200 ms (short preview condition) and when the preview time was lengthened to around 2000 ms (the long preview condition). In contrast, they only found more fixations on the phonological competitors than the distractors in the long preview condition, suggesting the phonological effect was less robust. Based on their findings, the authors concluded that both semantic and phonological information could guide eye movements to printed Chinese words during spoken word recognition, and the printed-word VWP may be a promising tool for investigating semantic information processing during Chinese spoken word recognition.

The main purpose of the current study is to find out how Chinese puns are processed. Specifically, in what manner does a pun receiver get the two intended meanings, simultaneously or sequentially? To answer this question, we have borrowed Shen and Li’s (2016) paradigm to test the predictions on this process made by DAM and GSH. According to DAM, the two context-compatible meanings of the ambiguous word in a pun are accessed at the same time, which should result in no fixation proportion difference to the two semantic competitors in the current experiment. However, GSH predicts that the salient meaning of the ambiguous word will be accessed first, leading to more selective attention to the semantic competitor related to the salient meaning.

## Materials and Methods

The experiment consisted of two experimental conditions and was conducted in the same session. The first condition (hereafter referred to as the **replication condition**) was designed to replicate the semantic effect reported by Shen et al. (2016) using the printed-word VWP. The second condition (hereafter referred to as the **pun condition**) was designed to examine the role played by salience in Chinese pun comprehension.

### Participants

Twenty-seven native Chinese speakers (17 males and 10 females, mean age = 21.2, *SD* = 2.0) participated in the experiment. They were students studying at a key university in China and had normal or corrected-to-normal vision. Participants were recruited through the campus forum and were paid a small amount of money after the experiment.

### Materials

Forty unambiguous sentences were selected as the auditory stimuli as the replication condition, most of which were chosen from Chinese newspaper headlines covering topics of education, business, health, etc. One target word was chosen from each sentence, which occurred near the end of the sentence. For each target word, four disyllabic words were chosen, including a semantic competitor, a phonological competitor, and two unrelated distractors. In order to avoid any confounding effect that may arise from phonological factors, all the semantic competitors (including those used in the pun condition) were strictly controlled so that they share no similar syllables with the spoken target word.

Forty pun sentences were selected from the same sources for the replication condition. They were selected in such a way that both meanings of the ambiguous words were supported by the pun context. For example, in the pun sentence 中意牌空调，您终生无憾/无汗的选择 (literal translation: *Zhongyi Brand air conditioner, your lifetime no sweat/regret choice*), the “no sweat” meaning is primed by the context word *air conditioner* while the “no regret” meaning is supported by the other context word *lifetime*. We selected puns in such a way to ensure that the participants would get the majority of the puns. More importantly, we also hoped to minimize the possible confounding effect that may arise from a strong contextual bias so that the possible salience effect could be better observed.

**FIGURE 1:**
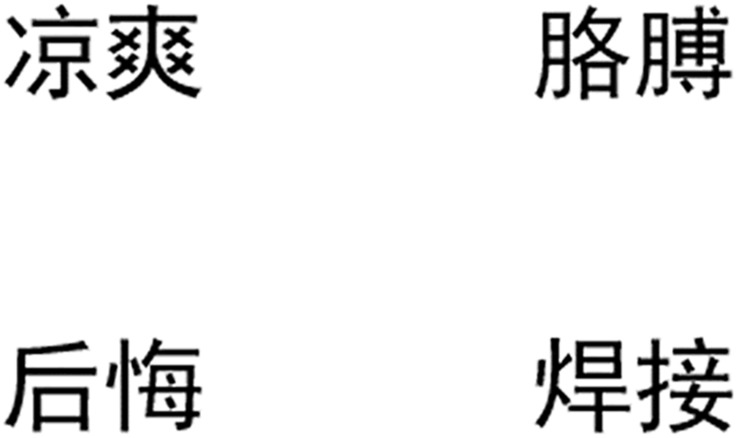
Example of a visual display used in the pun condition. For a pun sentence like “**中意牌空调您终生无汗/憾的选择**” (literal translation: Zhongyi Brand air conditioner, your lifetime no sweat/regret choice), the printed-word display consists of four conditions of the semantic competitor 1 **后悔** (*regret*/hou4 hui3/), the semantic competitor 2 **凉爽** (*coolness*,/liang2 shuang3/), the phonological competitor **焊接** (*welding*,/han4 jie1/), and the distractor **胳膊** (arm,/ge1 bo1/) in four different positions.

For the visual display, two semantic competitors were chosen in this condition: one is semantically related to the salient meaning of the ambiguous word in the pun (hereafter referred to as **semantic competitor 1**) and the other to the less salient meaning (hereafter referred to as **semantic competitor 2**). See the pretest in the “Materials and Methods” section for details on how the saliency of different meanings was decided. Besides, one phonological competitor and one distractor were chosen for each pun instead of two distractors. See Figure 1 for the visual stimuli used in the pun example. The complete lists of the visual stimuli used in both experiment conditions are also available in Supplementary Table S1.

The word frequency (Cai and Brysbaert, 2010) and the total number of strokes of the displayed words were carefully matched in both experimental conditions (*F*s < 1.1). See Table 1 for the lexical properties of the visual stimuli. To prevent the participants from realizing the manipulations between the spoken target words and the visually presented words, 80 filler trials were created in a similar fashion with the trials in the replication condition. However, none of the four displayed words are semantically related to the spoken target words in the filler trials. All the sentences used in the experiment were recorded at an average speaking rate by a female native Chinese speaker in a sound-proof booth with a sampling rate at 44.1 kHz.

**TABLE 1:** Properties of the visual stimuli used in both experimental conditions.

Experimental condition	Competitor type	Mean word frequency	Mean stroke number
Replication	Semantic Competitor	28.94	15.00
	Phonological Competitor	35.23	16.00
	Distractor 1	33.90	16.50
	Distractor 2	47.46	16.18
Pun	Semantic Competitor 1	39.82	15.25
	Semantic Competitor 2	37.87	16.53
	Phonological Competitor	33.05	16.30
	Distractor	47.98	15.18

*Word frequency is measured in occurrences per million.*

### Pretest to Measure the Saliency of the Two Meanings of a Lexically Ambiguous Word

One of the primary goals of the current study is to investigate the possible salience effect in pun comprehension. To decide which meaning associated with the ambiguous words in the pun condition is more salient, another group of 28 students from the same population (who did not participate in the eye-tracking experiment) was recruited to conduct a cross-modal lexical decision test (McKoon et al., 1996). In this test, the participants were required to make a lexical judgment as fast as possible on a visually presented 2-character Chinese word/non-word after hearing a spoken word. The forty ambiguous words (the prime) and their corresponding two semantic competitors (the probe) hence generated 80 trials and were separated into two lists so that the same prime would not appear on the same list. Moreover, 80 filler trials (60 non-word trials) were added into each list to balance the response. During the test, the trials in each list were presented to the participants in a pseudo-random fashion, and the order of the two lists was counterbalanced between participants.

Based on the test results, we defined the semantic competitors with shorter response times as related to the salient meaning (**semantic competitor 1**) and the ones with longer response times as related to the less salient meaning (**semantic competitor 2**). Statistic results show that the group of semantic competitor 1 (*M* = 599.79 ms, *SD* = 41.93) was responded much faster than the group of semantic competitor 2 [*M* = 659.94 ms, *SD* = 50.36, *t*_(__39__)_ = −8.08, *p* < 0.001].

### Normative Data on the Semantic Relatedness of the Competitors

Twenty-four raters who did not participate in the experiment were recruited to rate the semantic relatedness between the spoken target word (presented in Chinese pinyin) and the four printed-word stimuli on a 5-point Linkert scale (1 for very unrelated and 5 for very related). According to the ratings in the replication condition, the spoken target words were rated significantly more related to the semantic competitors (*M* = 4.44, *SD* = 0.32) than to either the phonological competitors [*M* = 1.45, *SD* = 0.23, *t*_(__39__)_ = 38.99, *p* < 0.001] or each group of the distractors [*M* = 1.37, *SD* = 0.22, *t*_(__39__)_ = 41.30, *p* < 0.001; *M* = 1.37, *SD* = 0.24, *t*_(__39__)_ = 46.07, *p* < 0.001]. As for the pun condition, the spoken ambiguous words were rated more related to both the group of semantic competitor 1 (*M* = 3.78, *SD* = 0.56) and the group of semantic competitor 2 (*M* = 3.63, *SD* = 0.52) than to either the phonological competitors (*M* = 1.36, *SD* = 0.21) or the distractors [*M* = 1.31, *SD* = 0.19] (*p* < 0.001). While both groups of semantic competitor 1 and semantic competitor 2 were rated equally related to the ambiguous word (presented in Chinese pinyin) [*t*_(39)_ = 1.18, *p* = 0.25], indicating that the ambiguous words from the puns were not rated as having a strong bias against either the salient meaning or the less salient meaning.

### Apparatus

Eye movements were tracked using an SR Research Eyelink 1000 plus system at a sampling rate of 1000 Hz, and eye movement data were collected from the right eye only. The experiment was carried out on a 19-inch monitor (Dell P1917S) with a refresh rate of 75 Hz and a screen resolution of 1024^∗^768 pixels. A chin rest with forehead support was used to minimize head movements during the experiment. Sentence recordings were presented to the participants through a headphone.

### Procedures

**FIGURE 2:**
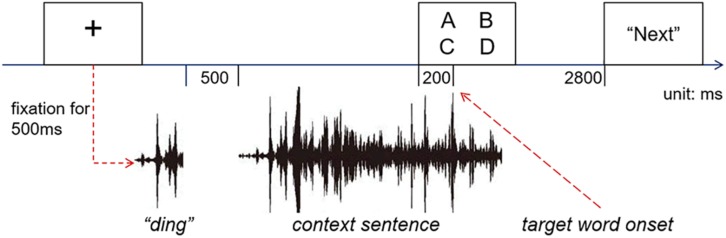
Trial structure of the experiment.

After the participants entered the lab, they were given a brief explanation about how eye trackers work as well as the instructions for the experiment. The participants were seated 60 cm from the video monitor. A nine-point calibration and validation procedure was used during the experiment, and the validation error was less than 1° of visual angle. At the beginning of each trial, a cross sign was displayed at the center of the screen. If no fixation was detected within the 1° of visual angle from the cross center within 5 s, the calibration procedure would be initiated again. Once the participants fixated at the cross for 500 ms, a “ding” sound was played, indicating the beginning of the spoken sentences. The cross mark stayed on the screen until it was replaced by a display of four words, 200 ms prior to the onset of the spoken target words^2^. The printed-words remained on the screen for 2800 ms to give participants sufficient time to comprehend the spoken sentence. All the printed-words were presented in 30-point Song font in white (RGB: 255, 255, 255) on a black background (RGB: 0, 0, 0). Each character subtended a visual angle of approximately 1.2°, and the center of each word is around 7.2° away from the screen center. The positions of the four visual words were counter-balanced, and all trials were presented pseudo-randomly so that the trials of the same experiment condition would be repeated no more than once. In each trial, the participants were asked to listen to the sentence carefully and to view the visually displayed words naturally without performing any explicit task. At the end of the trial, they were required to press the space bar to move on to the next trial. The trial structure is illustrated in Figure 2.

The participants were given six practice trials to familiarize themselves with the experimental procedure. The experimental trials were pseudo-randomly separated into four 40-trial blocks, and the participants were asked to take a short break after finishing each block. The entire experiment lasted around 40 min.

## Results

**FIGURE 3:**
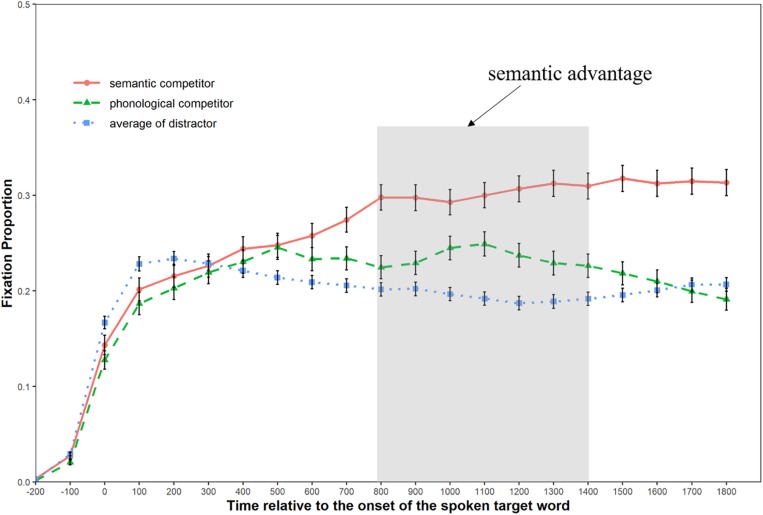
Graph showing the fixation patterns in the replication condition.

**FIGURE 4:**
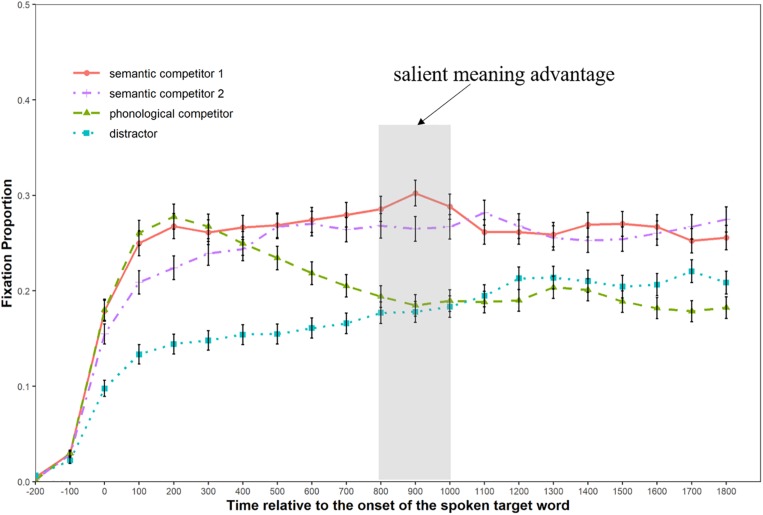
Graph showing the fixation patterns in the pun condition.

We defined fixation as a focus on the printed-word within a square of 5.5° × 5.5° visual angle centered at that word, and calculated the mean fixation proportions to the different types of printed-words (semantic competitors, phonological competitors, and distractors) in each experimental condition with a time bin of 100 ms, starting from the display onset of the printed words (200 ms prior to the target word onset) to 1800 ms afterward. Figures 3, 4 illustrate the fixation patterns to different competitor types in the replication condition and the pun condition, respectively.

As can be seen from Figure 3, the curve of fixation probability for the semantic competitor and the phonological competitor began to diverge from that of the distractor around 300 ms after the emergence of the spoken target word. Moreover, the curve of the semantic competitor started to separate from that of the phonological competitor 200 ms later (approximately equal to the offset of the target word). Figure 4 shows that the curve of fixation probability for the semantic competitor 1 started to diverge from that of the phonological competitor from about 300 ms after the onset of the target word. Meanwhile, the curve of fixation probability for the semantic competitor 2 caught up with the curve of phonological competitor 100 ms later and continued to rise till it intertwined with that of the semantic competitor 1 at the latter stage.

For the statistical analysis, two types of analysis were performed: the global analysis to capture the overall fixation pattern and the local analysis to detect possible subtle differences within a smaller time window. For the global analysis, two time-windows were chosen: an earlier one to analyze the fixation patterns during the unfolding of spoken target words and a latter one to capture the patterns after the target word offset. Since it should take approximately 150–200 ms to program and execute a saccade (Rayner, 1998), the first time window was set from 200–800 ms after the target word onset to examine the time period during the unfolding of the ambiguous word (the average duration of target words is around 560 ms) and the second one from 800 to 1400 ms to investigate the immediate time period after the target word was heard. In Coulson and Severens (2007)’s study, the salient meaning advantage disappeared when the ISI was prolonged to 500 ms, indicating that this advantage could only last for no more than 500 ms. However, without more laborious work (setting up experiment conditions with shorter ISIs, e.g., 400 or 300 ms), it would be challenging to narrow down the dominant meaning advantage much further. In the current paradigm, however, it was easier to time-lock this advantage to a more precise time window by using local analyses with shorter time-windows. For this purpose and in view of the 150–200 ms duration for eye saccades, three consecutive time-bins of 200 ms (800–1000 ms, 1000–1200 ms, and 1200–1400 ms) were chosen for the local analysis in the pun condition. Bonferroni correction was applied to the raw *p-*values to guard against the Type II error. Hence, only the adjusted *p*-values are reported in Tables 2, 3.

**TABLE 2:** Global analysis for competitor type of the two experimental conditions.

Condition	Time window (ms)	Predictor	Estimate	*SE*	Wald-*Z*	Adjusted *p*
Replication	200–800	(Intercept)^a^	–0.05	0.11	–0.49	0.624
		Semantic competitor	0.18	0.09	1.99	0.092
		Phonological competitor	0.12	0.09	1.34	0.180
	800–1400	(Intercept)^a^	–0.16	0.07	–2.33	0.020*
		Semantic competitor	0.73	0.11	6.71	< 0.001***
		Phonological competitor	0.27	0.09	2.95	0.003**
Pun	200–800	(Intercept)^b^	–0.51	0.09	–5.71	< 0.001***
		Semantic competitor 1	0.77	0.09	8.62	< 0.001***
		Semantic competitor 2	0.55	0.09	6.19	< 0.001***
		Phonological competitor	0.55	0.09	6.21	< 0.001***
	800–1400	(Intercept)^b^	–0.14	0.08	–1.83	0.067
		Semantic competitor 1	0.43	0.09	4.74	< 0.001***
		Semantic competitor 2	0.36	0.10	3.52	< 0.001***
		Phonological competitor	–0.28	0.10	–0.29	0.77

*p < 0.05, **p < 0.01, ***p < 0.001. ^*a*^One group of the distractors were randomly assigned as the baseline in the analysis. Since similar results were obtained, only one of them was reported. ^*b*^The distractor was assigned as the baseline for comparison.

**TABLE 3:** Local analysis for competitor type of the pun condition.

Time window (ms)	Predictor	Estimate	*SE*	Wald-*Z*	Adjusted *p*
800–1000	(Intercept)	–0.45	0.06	–7.22	< 0.001***
	Semantic competitor 2	–0.18	0.09	–2.05	0.04*
	Phonological competitor	–0.58	0.09	–6.17	< 0.001***
	Distractor	–0.65	0.09	–6.94	< 0.001***
1000–1200	(Intercept)	–0.55	0.06	–8.7	< 0.001***
	Semantic competitor 2	0.04	0.09	0.45	0.66
	Phonological competitor	–0.48	0.09	–5.09	< 0.001***
	Distractor	–0.43	0.09	–4.61	< 0.001***
1200–1400	(Intercept)	–0.64	0.06	–9.98	< 0.001***
	Semantic competitor 2	0.02	0.09	0.23	0.82
	Phonological competitor	–0.3	0.09	–3.2	0.03*
	Distractor	–0.45	0.09	–4.87	< 0.001***

*p < 0.05, **p < 0.01, ***p < 0.001. The semantic competitor 1 was assigned as the baseline for comparison.

Trials were excluded from analysis due to track losses, which accounted for 0.19% of all trials in the replication condition and 0.28% in the pun condition. The fixation proportion data were then analyzed using logit mixed-effect models (Ferreira et al., 2013). Compared with traditional analyses (e.g., *t*-test or ANOVA), the logit mixed effect model is more suitable for analyzing the categorical data in the present study, because the categorical outcomes violate several assumptions required by traditional analyses, such as the fact that the variances in two binomially distributed conditions should be homogeneous (Jaeger, 2008). In the analysis, we first defined the dependent variable as whether or not a fixation was detected in the four predefined interest areas within a specific time window, and the fixation count data was dummy-coded as “1” when the fixation was found in an interest area and as “0” when no fixation was detected. We then built logit mixed effect models in the R environment to fit the data (R Core Team, 2018) with the glmer function from the lme4 package (Bates et al., 2015). The competitor type (the semantic competitor, the phonological competitor, and the distractor) was defined as the fixed effect, and the participant and the trial item as the random factors. As suggested by Barr et al. (2013), we started the model with a maximum random effect structure, including a random intercept and slope for each participant and trial item. In the cases that the model failed to converge, the by-item random slop was dropped first, and then the by-participant random slop if necessary. Model comparison was performed using the ANOVA function to select the better model. The regression coefficient *b*, standard error (*SE*), and Wald-*Z* values are reported here.

Table 2 shows the global analysis results for both experimental conditions, where the distractor was used as the baseline to observe the possible phonological and semantic effect. In the replication condition, the semantic competitors received marginally more fixations than the distractor (*b* = 0.18, *SE* = 0.09, Wald-*Z* = 1.99, *p* = 0.09) during the 200–800 ms time window, while no fixation difference was found between the phonological competitors and the distractors (*p* = 0.18). In the 800–1400 ms time window, the semantic competitors received significantly more fixations than the distractors (*b* = 0.73, *SE* = 0.11, Wald-*Z* = 6.71, *p* < 0.001), while the phonological competitors also received more fixations than the distractors (*b* = 0.27, *SE* = 0.09, Wald-*Z* = 2.95, *p* < 0.001). In the pun condition, both types of semantic competitors and the phonological competitor received more fixations than the distractors in the 200–800 ms time window: the semantic competitor 1 (*b* = 0.77, *SE* = 0.09, Wald-*Z* = 8.62, *p* < 0.001), the semantic competitor 2 (*b* = 0.55, *SE* = 0.09, Wald-*Z* = −6.19, *p* < 0.001), and the phonological competitor (*b* = 0.55, *SE* = 0.09, Wald-*Z* = 6.21, *p* < 0.01). In the next 600 ms time window, the phonological competitor stopped to get more selective attention than the distractor (*p* = 0.77), while the semantic competitor 1 (*b* = 0.43, *SE* = 0.09, Wald-*Z* = 4.74, *p* < 0.001) and semantic competitor 2 continued to receive more fixations (*b* = 0.36, *SE* = 0.10, Wald-*Z* = 3.52, *p* < 0.001).

Table 3 shows the three consecutive time-bin analyses of the pun condition after the target word offset. Unlike the global analysis, the semantic competitor 1 was used as the baseline in the local analysis to investigate possible differences between the two types of semantic competitors. Consistent with the results from the global analysis (800–1400 ms), the analysis from the first time-bin (800–1000 ms) suggested that the semantic competitor 1 continued to receive more fixations than the distractor. More importantly, it also revealed that the semantic competitor 1 received more selective attention than the semantic competitor 2 (*b* = 0.18, *SE* = 0.09, Wald-*Z* = 2.05, *p* = 0.04) during this time window, approximately corresponding to the first 200 ms after the ambiguous words were fully heard. Such a difference between the two types of semantic competitors, however, was not detected in the next two time-bins (*p*s > 0.1).

## Discussion

The present study investigated whether the salient meaning of the ambiguous word in a pun is activated and accessed earlier than the other less salient meaning using a printed-word VWP. To test the validity of using such a paradigm in investigating the semantic processing of spoken Chinese pun, we used similar materials and procedures (the replication condition) as those in the study of Shen et al. (2016) and observed a reliable semantic effect, namely the effect that semantic competitors received more fixations than distractors. This result suggested that the semantic information of printed Chinese words was activated and utilized during spoken word recognition and thereby shifted the listeners’ attention. In the light of the proof, we felt more confident about the results from the pun condition. It was found that words semantically related to the salient meaning of the spoken target word (semantic competitor 1) received more fixations than the words semantically related to the less salient meaning (semantic competitor 2) within the first 200 ms after the target word is heard^3^, indicating a salient meaning advantage. In the next 400 ms, however, this difference in fixation proportion disappeared, suggesting both the salient and less salient meanings related to a pun became equally available to the participants.

### Validity of the Printed-Word VWP in Chinese Pun Research

One premise for the current study is that the printed-word VWP is valid for semantic research on Chinese puns. To clarify this, we need first to prove that the semantic effect reported in Shen et al.’s (2016) study is robust. Shen et al. (2016) showed that Chinese participants were sensitive to the semantic information in their printed-word VWP experiments, which contrasted with findings from participants with an alphabetic language background, such as English and Dutch (Huettig and McQueen, 2007; Salverda and Tanenhaus, 2010). These authors attributed their findings to the typological difference between alphabetic languages and logographic languages. Specifically, alphabetic languages like English have a stronger orthographic form-sound connection than logographic languages like Chinese, which have a stronger orthographic form-meaning relation. The successful replication of this semantic effect in both the replication and pun condition of the present study, therefore, has further proved its robustness. As a result, we feel more confident in using this paradigm for our pun investigation.

An interesting finding in the current study is that the phonological effect, previously reported in VWP research on alphabetic languages (e.g., Huettig and McQueen, 2007; Salverda and Tanenhaus, 2010), seemed less reliable when using printed Chinese words. In the replication condition, we failed to find a phonological effect in the earlier time window (200–800 ms), which was consistent with Shen et al. (2016)’s findings (the short-preview condition). However, these authors reported a phonological effect when the preview time was prolonged to around 2000 ms, which in the current study was revealed by the analysis in the later time window (800–1400 ms).

One possible reason for the less robust phonological effect is that the Chinese script bears a weak form-sound relationship insofar that more time is needed for Chinese readers to activate corresponding phonological representations in the mental lexicon. Previous studies have shown that the semantic information of printed Chinese words can be more efficiently retrieved by Chinese readers (Leck et al., 1995), while phonology may play a trivial role among skilled Chinese readers because of the opaque lexical level orthography-phonology mapping (Luo et al., 2018; Zhou et al., 2018). Moreover, in a functional magnetic resonance imaging study, Brennan et al. (2013) claimed that learning to read could reorganize the phonological awareness network only for alphabetic but not for logographic writing systems, which was attributed to the different principles for mapping between orthographic and phonological representations. As a result, the less robust phonological effect in the current study was most likely due to typological differences between the alphabetic and logographic writing systems.

Another evidence that the phonological effect is less stable in such a paradigm using Chinese can be seen from the reversed pattern in the pun condition: the phonological effect was found in the earlier time window but not the later one. This difference may partly result from the fact that the fixation probability of the distractor (the baseline) was lowered substantially when two semantic competitors were presented. As a result, the fixation difference between the phonological competitor and the distractor was enlarged even though the fixation probability on the phonological competitor was not significantly increased. See the section “Limitations and Suggestions for Future Studies” for the other possible explanation for this pattern.

### Salience Effect and Comprehension of Chinese Puns

Besides the semantic effect, we also observed a salience effect during the comprehension of Chinese puns in the current study, namely the pattern that the salient meaning associated with the homonym in a pun was invariantly activated first regardless of contextual bias. The GSH accommodates well the time course and the fixation data to the two groups of semantic competitors in the pun condition. In our experiment, the semantic competitor 1 attracted more fixations within 200 ms after the offset of the critical ambiguous word, indicating greater accessibility of the salient meaning of the ambiguous word. According to GSH, the salient meaning of a word/phrase is cognitively advantageous and will be more easily activated and accessed in the mental lexicon than the less salient ones. In a pun sentence, where both meanings are usually similarly supported as in the current experiment, GSH posits that the cognitive superiority of the salient meaning will not be obscured/masked by the context effect, and hence, accessed faster than the less-salient meaning. On the other hand, the current results are not compatible with the DAM, which predicts no systematic preference for a particular type of semantic competitors.

The current results differ from the findings reported by Coulson and Severens (2007) that both related meanings of a pun are immediately activated after it is heard, at least in the language-dominant left hemisphere. One possible explanation for this difference is that saliency in their study was decided based on the ratings of semantic relatedness between the probe word and the ambiguous word in the pun context. It is possible that some less salient meanings of a pun were rated as more related to the ambiguous word, hence masking the initial subtle difference in the activation of different meanings. In the current study, more fixations for the semantic competitor 1 (salient meaning) was only observed within 200 ms after the ambiguous word offset, indicating that this salient meaning advantage was short-lived. The finding that the fixation proportion for both semantic competitors did not differ in the following 400 ms in our experiment, on the other hand, is in general consistent with Coulson and Severens (2007)’s findings that when the inter-stimulus interval was prolonged to 500 ms, both meanings were found available in both hemispheres.

One implication from GSH for pun comprehension should be noted. According to this theory, though the salient meaning associated with a particular linguistic form is relative stable, it is still subjective to change based on personal experience. Compared with models relying on meaning frequency, GSH accounts for more individual variabilities. For example, when hearing the word *model*, readers of this paper may think of its “hypothesis” notion, while its “fashion” interpretation is more likely to come first to the mind of a costume designer. Therefore, a reasonable deduction from GSH is that pun comprehension is not a unified experience among different individuals. Instead, this experience is guided by the saliency of the different meanings associated with a word form or sound in a particular listener’s or reader’s mind. Despite the subtle initial difference owing to individual variabilities, however, access to both meanings are still required for the appreciation of humor created by puns.

### Salience Effect in a Less-Salient-Meaning-Biasing Context

It is worth mentioning that GSH does not deny the contextual effect but assumes that it works in parallel with the lexical module. According to GSH, in the situations where the less salient meaning is strongly supported by the context, the less salient meaning may seem to be accessed earlier than the salient meaning, but it merely results from the top–down prediction mechanism. This mechanism works in parallel with the mental lexicon module and cannot prevent the salient meaning from being activated first on encountering the word stimulus. For example, in the pun sentence “An old lady in the bank told me to check her balance, so I pushed her over,” it seems that one could directly arrive at the less salient interpretation (the amount of money in an account) for *balance* before the salient interpretation (being upright and steady) is evoked by the punchline “*so I pushed her over”^4^*. However, this does not mean that the silence effect could be surpassed. It is more likely that the initial less salient interpretation is mostly prediction-driven (based on inferences) instead of stimulus-driven (based on lexical meaning retrieval). On the other hand, the salient meaning should still be activated first (salience effect) when the word *balance* is encountered, which is then “put onto the back burner” by the context *bank.* Peleg et al. (2001) showed that contextual facilitation could occur even before the lexical stimulus was met, especially when the word appeared near the ending position of a sentence, fostering an impression of a selective process.

Current findings are also in general agreement with the reordered access model. Like GSH, this model also claims that the different meanings of an ambiguous word are accessed exhaustively and in an ordered fashion, in line with the modular view. In contrast with GSH, however, the reordered access model claims that this order is no fixed but subjective to contextual influence from very early on, which is supportive of an interactionist point of view (Peleg et al., 2001; Giora, 2003). According to the reordered access model, the dominant (more frequent) meaning of an ambiguous word is accessed faster than the subordinate (less frequent) ones. However, access to the subordinate meaning can be advanced (reordered) to such an extent that it could compete with the frequency advantage of the dominant meaning. This idea has been frequently used to explain the so-called subordinate-biased effect (Duffy et al., 1988, 2001). In the case of a pun, where the two meanings are similarly supported, this model also predicts that the salient (dominant) meaning will be accessed first, then followed by the less salient (subordinate) meaning (Sheridan et al., 2009; Dholakia et al., 2016).

Nevertheless, we are more inclined to agree with the GSH model. In a recent ERP study on the three-character verb-object metaphors by our lab, we manipulated the preceding lexical primes to bias either the literal or metaphorical meaning of the metaphor targets, which were rated metaphorically salient (Wang et al., 2018). Surprisingly, we found no significant difference in the N400 priming effect between the two priming conditions compared with an unrelated prime word. This finding indicates that the meaning salience of a word is immune to the contextual effect, at least in terms of a lexical context, supporting the modular view of GSH.

### Limitations and Suggestions for Future Studies

Two limitations of the current study have to be noted. Though the semantic relatedness was controlled carefully to ensure both phonological competitors and distractors to be unrelated with target words, their semantic relatedness with the preceding context words was controlled based on the authors’ linguistic knowledge. This variation may also partly explain why the fixation proportion on distractors at 0 ms differed in the two experimental conditions: in Figure 3, the fixation proportion curve of the distractor is higher than that of the phonological while the opposite pattern is observed in Figure 4. Shen et al. (2018) demonstrated an improved version of the printed-word VWP, in which they did not use any context sentence but the target word alone. Though this method could overcome the problem stated here, it does not apply to our study since the majority of puns works on the basis of a sentence context. In addition, the present study does not include a condition where the less salient meaning of the ambiguous word in the pun is favored. Although this does not affect the observation of the salience effect during Chinese pun comprehension, it is beneficial for future studies to incorporate this type of puns and get a clearer picture of how the salience effect (lexical-level input) may interact with the contextual effect (discourse-level information).

In the present study, we adopted the method of VWP and studied Chinese puns presented in their spoken form. However, due to the widespread homophones in Chinese, it is quite common to see homophone puns in Chinese newspapers, advertisements, and other written media. An interesting question remains that which of the homophone-pair to present could lead to a better pun effect. Jared and Bainbridge (2017) conducted an investigation in this direction by comparing the eye movement pattern while participants read homophone-error sentences (e.g., *The lawyer was very glad we could meat up*) and homophone-correct sentences (e.g., *The butcher was very glad to chop meat up for the stew*). Their findings showed that appreciation of the funniness of a pun was most strongly related to the strength of the association between the homophone and the critical context word (e.g., *butcher*). As mentioned in the previous discussion, Chinese is a logographic language, which has a stronger form-meaning relation than alphabetic languages. Further research on visually presented Chinese homophone puns may be beneficial for a more comprehensive understanding of pun processing mechanisms.

In summary, pun comprehension is modulated by the saliency of the two different meanings associated with the critical ambiguous word/sound, and pun experience may vary with the cognitive status of listeners. Besides, the current findings lend further support to the validity of using the printed-word visual world paradigm for semantic research on Chinese.

## Data Availability Statement

The datasets generated for this study are available on request to the corresponding author.

## Ethics Statement

The studies involving human participants were reviewed and approved by School of International Studies Zhejiang University. The patients/participants provided their written informed consent to participate in this study.

## Author Contributions

WZ, YW, and XW conceived and designed the experiments. WZ and YW performed the experiments. WZ analyzed the data and wrote the manuscript. WZ and XW revised the manuscript.

## Conflict of Interest

The authors declare that the research was conducted in the absence of any commercial or financial relationships that could be construed as a potential conflict of interest.

## Footnotes

1 The N400 component, a centroparietal negativity, is known to be sensitive to semantic violations or anomaly (Kutas and Hillyard, 1980). It is also commonly used as an index for semantic priming, with smaller N400 component indicating larger priming effect (Kutas and Federmeier, 2011).

2 The semantic effect was found in both the short-preview (200 ms) and long-preview (around 2000 ms) conditions of Shen et al. (2016)’s study. Therefore, only a short-preview time was used in the current study.

3 To be precise, this finding was captured in the 800–1000 ms time window, which is around 200–400 ms after the target word offset. However, for easier comparison with other studies, the results reported here has already taken the time need for a saccade (150–200 ms) into consideration.

4 For some individuals, e.g., gymnasts or acrobat, the meaning of “being steady” could be so salient that they may not even derive the “money” interpretation before its further suppressed by the punch line.
